# A Gallstone Masquerading as Hepatic Decompensation: A Case of Bouveret Syndrome

**DOI:** 10.7759/cureus.116722

**Published:** 2026-09-22

**Authors:** Likhitha Nadendla, Shanthi Vijayaraghavan

**Affiliations:** 1 Hepatology, Sri Ramachandra Institute of Higher Education and Research, Chennai, IND

**Keywords:** alcohol-related liver disease, bouveret syndrome, chronic liver disease, gallstone ileus, gastric outlet obstruction

## Abstract

Bouveret syndrome is a rare form of gallstone ileus caused by impaction of a gallstone in the pylorus or proximal duodenum after migration through a bilioenteric fistula. Its nonspecific presentation can mimic hepatic decompensation in patients with chronic liver disease. We report the case of a 55-year-old man with alcohol-related liver disease who presented with jaundice, abdominal distension, abdominal pain, constipation, and bilious vomiting, with renal dysfunction and ascites. Contrast-enhanced CT showed acute calculous cholecystitis with sealed-off gallbladder perforation, a probable cholecystoenteric fistula, and a 25-mm gallstone impacted in the second part of the duodenum causing gastric outlet obstruction. The patient was managed conservatively in view of high surgical risk. He improved clinically, and repeat CT demonstrated spontaneous passage of the gallstone. This was a rare case of abdominal pain and jaundice in a patient with decompensated chronic liver disease. Early cross-sectional imaging is important for diagnosis and individualized management.

## Introduction

Bouveret syndrome is a rare variant of gallstone ileus in which a gallstone passes through a bilioenteric fistula and becomes impacted in the pylorus or proximal duodenum, causing gastric outlet obstruction. Gallstone ileus complicates approximately 0.3-0.5% of patients with cholelithiasis, while Bouveret syndrome represents a small proportion of gallstone ileus cases and accounts for fewer than 5% of gallstone ileus cases [1-4]. It generally results from chronic cholelithiasis and recurrent inflammation, leading to adhesions and fistula formation between the gallbladder and adjacent bowel, most commonly the duodenum. A large stone may then migrate through the fistula and cause obstruction [2-4]. Though Bouveret syndrome predominantly occurs in elderly patients with multiple comorbidities, it can occur outside this typical age group. Although rare, Bouveret syndrome is associated with substantial morbidity and mortality, with reported mortality rates of approximately 12-30%, particularly because of delayed diagnosis, gastric outlet obstruction, and the presence of significant comorbidities [5].

We report a case of Bouveret syndrome mimicking hepatic decompensation to highlight the importance of early cross-sectional imaging in patients with chronic liver disease and atypical gastrointestinal symptoms. The patient had alcohol-related liver disease and presented with gastrointestinal symptoms such as nausea, vomiting, and abdominal pain, which are nonspecific and may overlap with manifestations of hepatic decompensation, making the diagnosis challenging. The classic Rigler's triad consists of pneumobilia, an ectopic gallstone, and intestinal obstruction; however, all three features may not be present, and cross-sectional imaging is required for diagnosis. Spontaneous passage of an impacted gallstone caused Bouveret syndrome in this patient, which is uncommon and represents a distinctive feature of this case.

## Case presentation

A 55-year-old man with known compensated alcohol-related chronic liver disease with a baseline Child-Pugh score [6] of 7/B and MELD-Na (Model for End-Stage Liver Disease-Sodium) score [7] of 9 presented to the emergency room with an acute two-day history of jaundice, abdominal distension, diffuse abdominal pain, constipation, and bilious vomiting. He had no previous history of gallstones or abdominal surgery. On presentation, he was afebrile (temperature 98.6°F), icteric, and had bilateral pedal edema. Abdominal examination showed free fluid, diffuse tenderness, and sluggish bowel sounds. Vital signs showed tachycardia (pulse rate of 110 beats/minute), tachypnea (respiratory rate of 28 breaths/minute), and hypotension (blood pressure 90/60 mmHg). 

Laboratory investigations demonstrated leukocytosis, elevated inflammatory markers, cholestatic hepatitis on liver function tests, hypoalbuminemia, prolonged international normalized ratio (INR), elevated serum creatinine, and hyponatremia (Table 1). Blood culture and urine culture were sterile. Diagnostic paracentesis demonstrated a markedly high serum ascites albumin gradient (SAAG), neutrocytic ascites, elevated protein, low glucose, and polymicrobial growth in culture, consistent with secondary bacterial peritonitis according to Runyon's criteria (Table 2)

**Table 1:** Blood investigations INR: international normalised ratio; AST: aspartate aminotransferase; ALT: alanine aminotransferase; ALP: alkaline phosphatase; GGT: gamma-glutamyl transferase; LDH: lactate dehydrogenase; CRP: C-reactive protein

Investigations	Patient Value	Reference Range	
Hemoglobin	12.2 g/dL	12-15 g/dl	
Leukocyte count	19980 cells/µL	4000-11,000 cells/µL	
Platelets	1.56 lakhs/µL	1.5-4.5 lakhs/µL	
INR	1.75	0.8-1.2	
Total Bilirubin	28.44 mg/dL	0.2-1.2 mg/dl	
Direct Bilirubin	22.4 mg/dL	0.02-0.3 mg/dL	
AST	176 U/L	10-40 U/L	
ALT	96 U/L	7-40 U/L	
ALP	137 U/L	44-147 U/L	
GGT	80 U/L	9-40 U/L	
Albumin	2.6 g/dL	3.5-5 g/dL	
Creatinine	2.4 mg/dL	0.7-1.2 mg/dL	
Sodium	128 mmol/L	135-145 mmol/L	
Potassium	5 mmol/L	3.5-5 mmol/L	
Chloride	98 mmol/L	98-106 mmol/L	
LDH	140 U/L	140-280 U/L	
CRP	15 mg/dL	0.3-1 mg/dL	

**Table 2:** Ascitic fluid analysis LDH: lactate dehydrogenase; ADA: adenosine deaminase; PMN: polymorphonuclear leucocyte

Ascitic Fluid Analysis	Patient Value	Reference Range	
Total protein	2.6 g/dL	2.5-3 g/dL	
Albumin	1.4 g/dl	-	
Glucose	40 mg/dL	70-100 mg/dL	
LDH	145 U/L	100-200 U/L	
ADA	27.9 U/L	30-40 U/L	
PMN	124 LL cells/mm³	<250 cells/mm³	
Culture	Polymicrobial		

Contrast-enhanced CT (CECT) demonstrated acute calculous cholecystitis with sealed-off gallbladder perforation, a probable cholecystoenteric fistula, and a 25-mm gallstone in the second part of the duodenum with gastric outlet obstruction, consistent with Bouveret syndrome. Features suggestive of chronic liver disease with portal hypertension and moderate ascites were also noted (Figure 1). 

**Figure 1:**
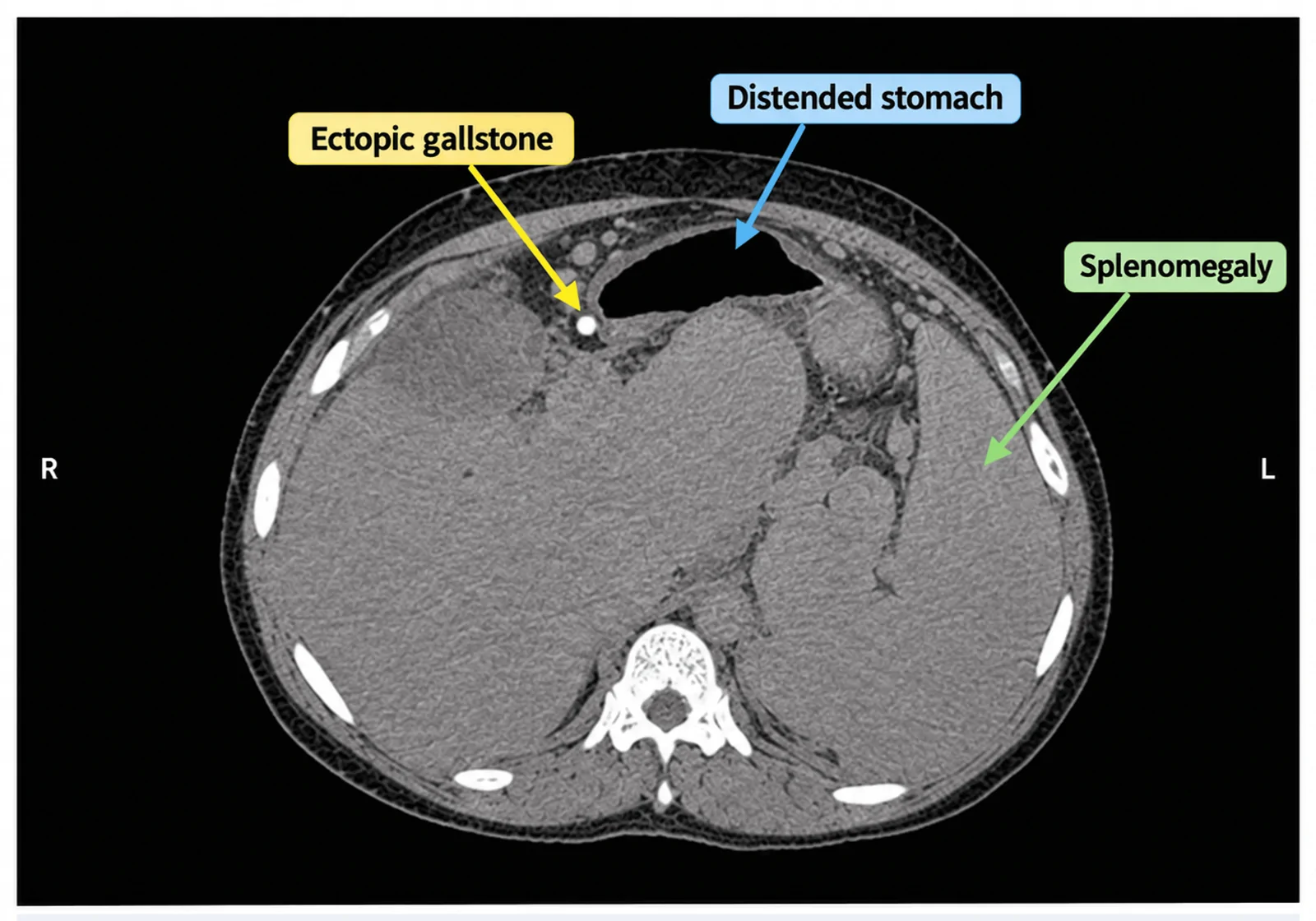
Contrast-enhanced CT scan of the abdomen (axial view) demonstrating features of Bouveret syndrome Image shows a distended stomach containing gas and fluid (blue arrow) and a hyperdense ectopic gallstone within the second part of the duodenum (yellow arrow), with associated features of chronic liver disease and splenomegaly (green arrow).

In view of decompensated liver disease and high surgical risk (Child-Pugh score of 12/C, MELD-Na 36, American Society of Anesthesiologists (ASA) 4E), the patient was managed conservatively with broad-spectrum antibiotics (meropenem) for seven days and supportive care, which included intravenous fluids, intravenous albumin, electrolyte monitoring and correction, antiemetics, nutritional support, and standard management of chronic liver disease.

By day 3 of conservative management, the patient's abdominal pain, vomiting, and constipation had resolved. His acute kidney injury also improved (serum creatinine decreased from 2.4 to 1.8 mg/dL, and serum sodium increased from 128 to 131 mmol/L). Oral intake was gradually reintroduced and was well tolerated. 

A repeat CT showed persistent cholelithiasis with hemorrhagic cholecystitis, but the previously visualized duodenal gallstone was no longer seen, with improvement of previously demonstrated gastric outlet obstruction, suggesting spontaneous passage of the stone (Figure 2). Magnetic resonance cholangiopancreatography (MRCP) confirmed cholelithiasis with hemorrhagic cholecystitis. As the patient's clinical condition continued to improve, endoscopic/surgical intervention was not considered, and he was successfully managed non-operatively.

**Figure 2:**
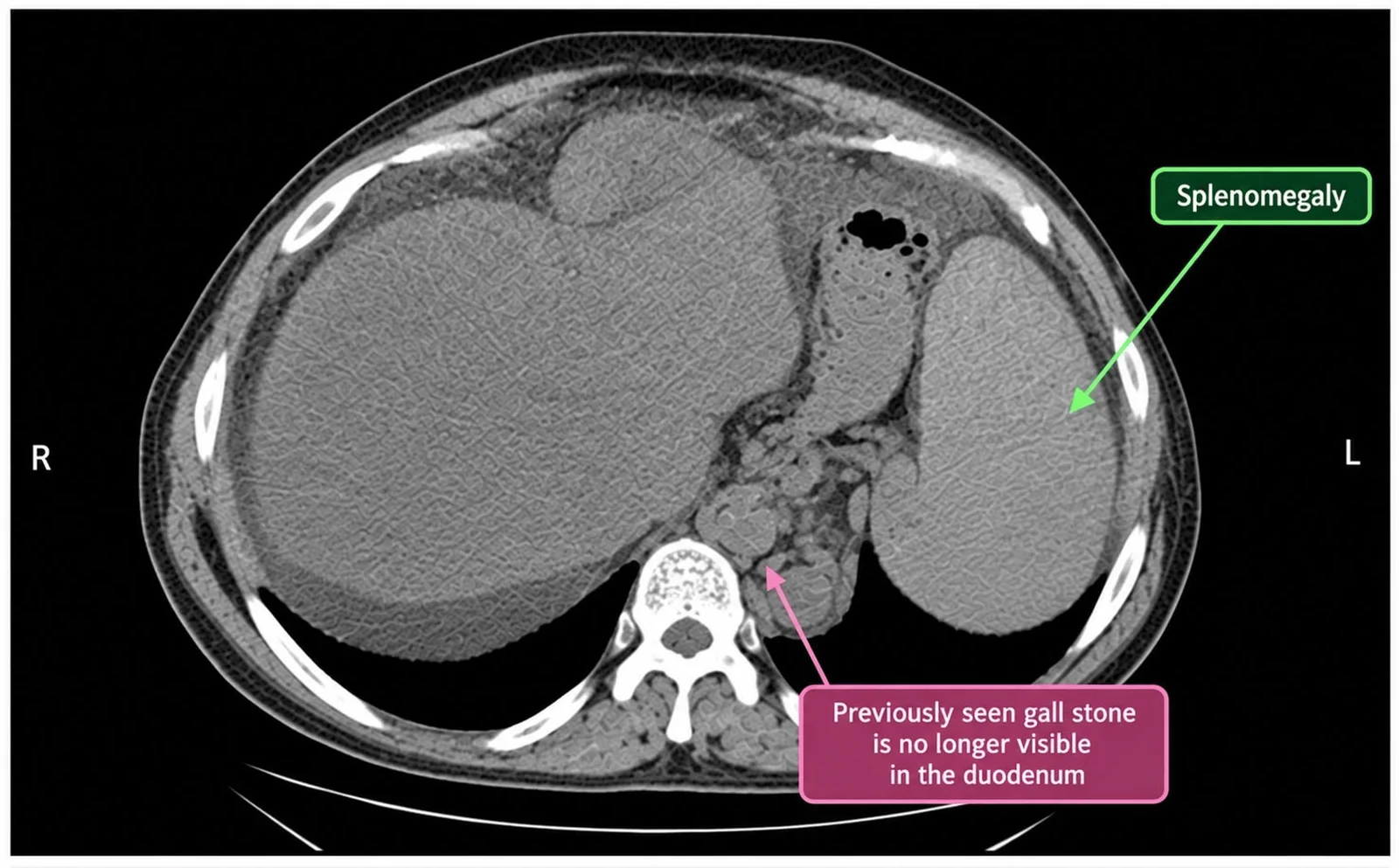
Repeat contrast-enhanced CT of the abdomen (axial view) Image shows splenomegaly (green arrow) and the previously seen hyperdense focus (ectopic gallstone) is no longer visible in the duodenum (pink arrow).

At the two-week and four-week follow-ups, the patient remained clinically well without recurrent vomiting, abdominal pain, or features of intestinal obstruction.

## Discussion

Bouveret syndrome is an uncommon and underrecognized cause of gastric outlet obstruction. It develops when a gallstone migrates through a bilioenteric fistula, usually cholecystoduodenal, and becomes impacted in the proximal gastrointestinal tract [1-4]. Diagnosis may be difficult because vomiting, abdominal pain, jaundice, ascites, and renal dysfunction can be attributed to hepatic decompensation in patients with chronic liver disease.

The pathophysiology typically involves chronic inflammation from recurrent cholelithiasis, leading to adhesions between the gallbladder and adjacent duodenum or stomach. Persistent inflammation and pressure necrosis from a large gallstone can result in erosion of the intervening tissues and formation of a cholecystoenteric fistula. Through this fistulous communication, a sufficiently large gallstone may enter the gastrointestinal tract and become impacted, most commonly in the duodenal bulb or pyloric region, producing gastric outlet obstruction. The risk of obstruction is influenced by stone size, local anatomy, and the degree of luminal narrowing [1-4]. In this case, bilious vomiting and constipation prompted further evaluation despite the apparent features of hepatic decompensation. CECT was diagnostic, demonstrating the ectopic duodenal stone, gastric outlet obstruction, and probable cholecystoenteric fistula. CT is particularly useful because it can identify the ectopic stone, obstruction, fistula, and associated hepatobiliary complications. Although Rigler's triad of pneumobilia, ectopic gallstone, and intestinal obstruction is classically described, all components need not be present [8]. MRCP can further characterize the biliary system and associated inflammatory pathology. Management should be individualized and may include endoscopic, surgical, or conservative treatment. Endoscopic extraction or lithotripsy can avoid surgery but may be limited by stone size, location, and impaction. Surgery is definitive but carries substantial perioperative risk in patients with advanced liver disease [1,3]. In our patient, in view of underlying liver disease, conservative management was chosen. Clinical improvement and disappearance of the stone on repeat imaging suggested spontaneous passage. Spontaneous passage of an impacted stone in Bouveret syndrome is uncommon. This case suggests that carefully selected high-risk patients who improve clinically may be considered for non-operative management with close monitoring. The key lesson is that gastrointestinal symptoms in patients with chronic liver disease should not automatically be attributed to hepatic decompensation. Persistent vomiting, constipation, or atypical abdominal findings should prompt evaluation for mechanical pathology, with early cross-sectional imaging when appropriate.

## Conclusions

Bouveret syndrome is a rare cause of gastric outlet obstruction that can mimic hepatic decompensation in patients with alcohol-related liver disease. Persistent vomiting and obstructive symptoms should prompt consideration of mechanical pathology and early cross-sectional imaging, particularly contrast-enhanced CT when appropriate, which can facilitate recognition of mechanical gastrointestinal pathology and guide individualized management. In this case, non-operative management was associated with clinical improvement and apparent spontaneous passage of the impacted gallstone. 

Although this case suggests that conservative management may be an option in carefully selected high-risk patients, this observation is based on a single case and should not be generalized. Endoscopic or surgical removal of the obstructing stone remains the principal therapeutic approach for most patients, while the choice of intervention should be individualized according to the patient's comorbidities, clinical status, and procedural risk.
